# Recent Advances in Diagnosing Chronic Pulmonary Aspergillosis

**DOI:** 10.3389/fmicb.2018.01810

**Published:** 2018-08-17

**Authors:** Takahiro Takazono, Koichi Izumikawa

**Affiliations:** ^1^Department of Infectious Diseases, Graduate School of Biomedical Sciences, Nagasaki University, Nagasaki, Japan; ^2^Second Department of Internal Medicine, Nagasaki University Hospital, Nagasaki, Japan

**Keywords:** aspergillosis, *Aspergillus*, galactomannan, *Aspergillus* IgG antibody, azole resistance

## Abstract

**Purpose:** The diagnosis of chronic pulmonary aspergillosis (CPA) is occasionally complicated due to poor sensitivity of mycological culture and colonization of *Aspergillus* species in the airway. Several diagnostic methods have been developed for the diagnosis of invasive pulmonary aspergillosis; however, their interpretation and significance are different in CPA. This study aimed to review the recent advances in diagnostic methods and their characteristics in the diagnosis of CPA.

**Recent findings:** Radiological findings of lung, histopathology, and culture are the gold standard of CPA diagnosis. Serodiagnosis methods involving the use of galactomannan and β-D-glucan have low sensitivity and specificity. An *Aspergillus*-specific IgG antibody assay showed good performance and had better sensitivity and reproducibility than conventional precipitant antibody assays. Currently, it is the most reliable method for diagnosing CPA caused by *Aspergillus fumigatus*, but evidence on its effectiveness in diagnosing CPA caused by *non-fumigatus Aspergillus* is lacking. Newly developed lateral flow device Aspergillus and detection of volatile organic compounds in breath have potential, but evidence on its effectiveness in diagnosing CPA is lacking. The increasing prevalence of azole-resistant *A. fumigatus* strains has become a threat to public health. Some of the azole-resistant-related genes can be detected directly from clinical samples using a commercially available kit. However, its clinical efficacy for routine use remains unclear, since resistance-related genes greatly differ among regions and countries.

**Conclusion:** Several issues surrounding the diagnosis of CPA remain unclear. Hence, further investigations and clinical studies are needed to improve the accuracy and efficiency of CPA diagnosis.

## Introduction

*Aspergillus* species are environmental molds that produce airborne spores, and the average human is estimated to inhale hundreds of *Aspergillus* conidia daily (Hospenthal et al., 1998). Host immunity and the underlying pulmonary diseases are critical factors in determining the outcome of this daily exposure. Patients with defects in cell-mediated immunity, including those with neutropenia due to cytotoxic chemotherapy, or T-cell dysfunction due to corticosteroid or other immunosuppressive therapy are at risk of developing invasive pulmonary aspergillosis (IPA) characterized by hyphal invasion of lung tissues and dissemination to other organs (Baddley, 2011; Patterson et al., 2016). However, patients with underlying chronic respiratory disorders, such as chronic obstructive pulmonary disease, post-pulmonary tuberculosis, non-tuberculosis mycobacteriosis (NTM), cystic fibrosis (CF), bronchiectasis, or allergic bronchopulmonary aspergillosis could develop saprophytic *Aspergillus* colonization and infection, namely, chronic pulmonary aspergillosis (CPA) (Saraceno et al., 1997; Takeda et al., 2016; Lowes et al., 2017). CPA is a slowly progressive pulmonary disease caused by *Aspergillus spp*. (Saraceno et al., 1997) and its prognosis is poor; the 5-year mortality rate of CPA patients is approximately 50–85% (Lowes et al., 2017). CPA is categorized into five disease entities based on the recent guidelines of the European Respiratory Society: *Aspergillus* nodule, simple pulmonary aspergilloma, chronic cavitary pulmonary aspergillosis (CCPA), chronic fibrosing pulmonary aspergillosis (CFPA), and subacute invasive pulmonary aspergillosis (SAIA) (Denning et al., 2016).

The diagnosis of CPA is occasionally complicated, as there are several disease entities in CPA, which are described in the following section, and some patients with underlying pulmonary diseases develop *Aspergillus airway colonization*. Diagnostic methods used for CPA are similar with those of IPA, but their interpretation and significance are different. Clinicians need various clinical information such as patients' background, radiological images, clinical courses, cultural tests, and other supportive diagnostic methods to diagnose CPA. The present review describes the currently available diagnostic methods and discusses new approaches for diagnosing CPA and their future directions.

### Radiological and histopathological findings

Simple pulmonary aspergilloma is defined as single pulmonary cavity containing a fungal ball in a non-immunocompromised patient with minor or no symptoms and no radiological progression over at least 3 months of observation. Aspergillus nodule is characterized by the presence of one or more nodules without cavitation caused by *Aspergillus spp*. (Denning et al., 2016; Muldoon et al., 2016).

On the contrary, CCPA and SAIA are characterized by one or more cavities with or without fungal ball and its radiological progression such as expanding thick-walled cavities and pericavitary infiltration (Denning et al., 2016). The crucial difference between them is that SAIA involves hyphal invasion into the lung parenchyma (Yousem, 1997; Hope et al., 2005); however, it is not occasionally easy and practical to obtain sufficient histopathological samples to confirm the diagnosis. Therefore, clinical information such as time course of radiological progression (CCPA >3 months; SAIA 1–3 months) and process of cavity formation are indispensable for clinical diagnosis; CCPA usually occurs in pre-existing cavities, whereas in SAIA, cavities can be subsequently formed by the necrotic change of nodules or infiltration lesion due to *Aspergillus* species. infection (Izumikawa et al., 2014). However, it is hard to distinguish them if the serial radiography films are not available. Particularly, the patients with NTM infection are difficult to diagnose due to their similarity in radiological findings such as nodular shadows and cavity formation (Kobashi et al., 2006). CFPA is defined as severe fibrotic destruction of at least two lung lobes complicating CCPA leading to a major loss of lung function and generally the end result of untreated CCPA (Denning et al., 2003, 2016). Thus, these three clinical entities are vague and overlapping in some cases; however, it is essential to distinguish them in order to estimate their prognoses. Although triazole antifungals are recommended in these entities, their efficacy was better in patients with SAIA than in those with CCPA, as reported in a prospective study in France (Cadranel et al., 2012). Recently, “scab-like sign” observed inside the cavitary lesion in CT was proposed as a high-risk sign of hemoptysis in CPA patients, this could be useful when following the CPA patients (Sato et al., 2018).

### Mycological culture

Mycological culture is the basic methods for diagnosing CPA, although it has several limitations. The culture positivity rates of *Aspergillus* species from respiratory specimens in CPA vary widely, ranging from 11.8 to 81.0% depending on reports (Kitasato et al., 2009; Kohno et al., 2010; Nam et al., 2010; Shin et al., 2014). Uffredi et al. reported that 48 (63%) individuals were colonized patients among 76 non-granulocytopenic patients whose respiratory specimens yielded *Aspergillus fumigatus* (Uffredi et al., 2003). In our previous study, only 11 (16.4%) of 67 individuals were colonized patients among those with culture positive for *A. fumigatus*. By contrast, 58 (65.9%) of 88 individuals were colonized patients whose cultures yielded *non-fumigatus Aspergillus* strains (Tashiro et al., 2011). These reports imply that the clinicians need to be careful when interpreting the results of fungal cultures from respiratory specimens, as *Aspergillus* species are ubiquitous organism that is present in the air, and some of them are saprophytic fungus and cannot be the target of treatment. The most important way to distinguish the colonization from infection is to confirm clinical information, such as the transitional change of radiological findings; however, films are not always available. Therefore, we need a biomarker that reflects the invasiveness of *Aspergillus* infection.

### Antigen and antibody test

It is not always easy to obtain the histopathological specimen, as some patients are not tolerable for invasive diagnostic procedure such as transbronchial lung biopsy due to their general conditions; therefore, serodiagnosis is indispensable for the diagnosis of CPA. Galactomannan (GM) antigen assays in serum and bronchial alveolar lavage (BAL) fluid have high sensitivity and specificity for the diagnosis of IPA, with cutoff values of 0.5 and 1.0, respectively (Maertens et al., 2007, 2009). However, the GM serum assay has lower sensitivity and specificity for CPA, with a cutoff value of 0.5 (Kitasato et al., 2009; Shin et al., 2014), than for IPA. GM antigen in BALF showed relatively higher sensitivity (77.2%) and specificity (77.0%), with a cutoff value of 0.4, than that in serum (Izumikawa et al., 2012).

Although the β-D-glucan (BDG) assay has high sensitivity for the screening of a wide range of invasive fungal infections such as candidemia, pneumocystis pneumonia, and IPA, its specificity is limited (Karageorgopoulos et al., 2011; Onishi et al., 2012). Furthermore, its sensitivity is very low (about 20%) in CPA patients (Kitasato et al., 2009; Kohno et al., 2010). Urabe et al. recently reported that the combination of GM and BDG assays in BALF had a higher diagnostic accuracy compared with other single or combinations of diagnostic methods including PCR (Urabe et al., 2017).

Detection of the *Aspergillus*-specific antibody plays an important role in the diagnosis of CPA and Allergic bronchopulmonary aspergillosis and this method has been widely used. The precipitating *Aspergillus* IgG antibody has better sensitivity (80–90%) than GM and BDG assays (Kitasato et al., 2009; Kohno et al., 2010) At the moment, commercial *Aspergillus*-specific IgG plate ELISA tests are currently produced by Serion (Germany), IBL (Germany/USA), Dynamiker/Bio-Enoche (China), Bio-Rad (France), Bordier (Switzerland), and Omega/Genesis (UK) (Page et al., 2015). Siemens (Germany) supplies an automated *Aspergillus-*specific IgG ELISA system (Immunolite), while Thermo Fisher Scientific/Phadia (multinational) supplies an automated *Aspergillus*-specific IgG fluoroenzyme immunoassay system (ImmunoCAP), which is an ELISA variant (Page et al., 2015). The Phadia ImmunoCAP IgG assay and Bio-Rad Platelia *Aspergillus* IgG method have been reported to possess better sensitivity and reproducibility compared with the method involving the use of the conventional precipitant antibody (Baxter et al., 2013). These detection kits have excellent performance in the diagnosis of CPA and ABPA (Baxter et al., 2013; Dumollard et al., 2016; Fujiuchi et al., 2016; Page et al., 2016, 2018). However, all these tests use purified antibodies to culture extracts or recombinant antigens of *A. fumigatus*, and were originally designed to detect *A. fumigatus*. As non-fumigatus strains account for 40% (30 of 74) of CPA patients in Japan (Tashiro et al., 2011) and 38% in India (Shahid et al., 2001), these assays might have limitations in diagnosing CPA caused by non-*fumigatus* strains in some areas.

### Polymerase chain reaction (PCR)

Polymerase chain reaction (PCR) for the diagnosis of IPA has been used for over 2 decades, though is not included in the European Organization for the Research and Treatment of Cancer/Mycoses Study Group (EORTC/MSG) definitions of invasive fungal disease (White et al., 2015). *Aspergillus* PCR from blood sample has similar sensitivity and specificity for the diagnosis of IPA (White et al., 2015), but failed to detect *Aspergillus* DNA in patients with SPA and CPA (Imbert et al., 2016), conversely, this implies that PCR could be useful to eliminate disseminated infection from CPA. In BALF sample, PCR showed tolerable sensitivity (66.7–86.7%) and specificity (84.2–94.2%) compared to GM or BDG (Urabe et al., 2017). RT-PCR has advantages, (1) its quantitative aspect offers the possibility to establish precise cutoff values that could distinguish colonization from active infections, (2) since RT-PCR detects RNA, which is an indicator of the living fungal cells.

### New strategies

*Aspergillus*-specific lateral flow device (LFD) was newly developed. It uses the mouse monoclonal antibody JF5, which binds to a protein epitope present on an extracellular glycoprotein antigen secreted constitutively during the active growth of *A. fumigatus*. This method can detect Aspergillus antigens in human serum within 15 min. An early clinical trial showed that LFD is comparable to GM in serum in terms of diagnosing IPA, with a sensitivity and specificity of 81.8 and 98%, respectively (White et al., 2013). In a single center prospective study, LFD test using BALF specimen also showed tolerable sensitivity (77%) and specificity (92%) for proven/probable IPA (Prattes et al., 2014). However, recently, a single center study reported that LFD showed low sensitivity of 38% for IPA (Castillo et al., 2018). The evidence of LFD's utility in CPA diagnosis is quite limited to date, clinical studies on the diagnosis of CPA are needed to better understand the clinical use of LFD.

Volatile organic compounds (VOCs) are known to be detected from the breath of an infected individual. Initially, 2-pentylfuran was reported as the potential diagnostic VOC in IPA patients (Syhre et al., 2008; Chambers et al., 2009). A recent proof-of-principle study was conducted using electronic noses to detect the characteristic VOC pattern of IPA and showed high sensitivity of 100% and a specificity of 83.3% (de Heer et al., 2013). Other researchers used thermal desorption-gas chromatography/mass spectrometry to detect the specific VOCs pattern of IPA and also showed high sensitivity of 94% and specificity of 93% (Koo et al., 2014). Moreover, Heer et al. applied the same methods to detect *A. fumigatus* colonization in CF patients and showed sensitivity of 78% and specificity of 94% (de Heer et al., 2016). These methods can be useful screening tests, as they are noninvasive diagnostic procedures; however, there might be an issue in distinguishing CPA from *Aspergillus*-colonized patients.

Galactosaminogalactan (GAG) is a newly discovered extracellular polysaccharide of *Aspergillus* species, composed of α-1-4-linked galactose and α-1-4-linked N-acetylgalactosamine. It was observed only in hyphae form (Fontaine et al., 2011). GAG is particularly abundant in *A. fumigatus*, which is the most pathogenic specie among hundreds of *Aspergillus* species (Lee et al., 2015). Furthermore, GAG is required for its virulence (Gravelat et al., 2013). Therefore, this component could be a potential biomarker to estimate the invasiveness of *Aspergillus* infection.

### Diagnosis of infection with azole-resistant *A. fumigatus*

In recent years, the global increase of azole-resistant *A. fumigatus* became an emerging concern for public health, despite the fact that the rates of resistant strains vary among regions, countries, or continents, and rates of resistant strains are especially high in European countries (van der Linden et al., 2015; Meis et al., 2016; Rivero-Menendez et al., 2016). Azole antifungals are the mainstay of treatments for pulmonary aspergillosis. The mortality rates in IPA patients infected with azole-resistant strains were higher than those infected with azole-sensitive ones (88% vs. 30–50%) (van der Linden et al., 2011). Lowes et al reported that the 10-year survival of CPA patients in United Kingdom with isolates fully susceptible to azoles was 68%, in contrast to 46% in patients with an isolate with reduced susceptibility to azoles, though there was not a significant difference (Lowes et al., 2017). However, it is still unclear how patients acquired azole-resistant strain infection affects the clinical course or mortality in CPA patients, because some azole-resistant strains obtained from aspergillosis patients treated with azoles showed poor condition and attenuated growth activity in *in vitro* condition (Ballard et al., 2018).

CPA patients need at least 6 months of oral azole treatment (Denning et al., 2016); detecting the azole-resistant strain earlier could provide them benefit by changing the treatment regimen. However, it is difficult to diagnose azole-resistant *A. fumigatus* infection in the clinical setting, as *in vitro* antifungal susceptibility testing of *Aspergillus* species is not routinely done in most clinical laboratories due to its cost and technical problems. The screening test with azole containing (itraconazole, 4 mg/L; voriconazole, 1 mg/L; posaconazole, 0.5 mg/L; and no antifungal) 4-well agar plate showed a sensitivity of 99% and a specificity of 99%, to screen the azole-resistant mutants (Arendrup et al., 2017); this could be useful and practical for routine test in clinical laboratories in countries where azole-resistance rate is high.

Azole-resistant *A. fumigatus* strains are mainly categorized into “environmental route” and “patient-acquired route” by means of resistance acquisition. The former was estimated to be generated by the agricultural fungicides used for crop protection and carries the tandem repeats (TR) of 34, 46, and 53 base pairs upstream in the promoter region of CYP51A with a single point mutation of CYP51A gene. By contrast, the latter were generated by the long-term use of medical azoles and carries various single point mutations of CYP51A gene (Meis et al., 2016). The environmentally obtained azole-resistant strains seemed to originate in Europe and have already spread into other regions worldwide (Meis et al., 2016).

The most commonly used method is simple polymerase chain reaction (PCR) amplification of the entire coding and promoter region with sequence analysis of the PCR products; however, this method is not practical for clinical use as it is time consuming. Restriction fragment length polymorphism by AluI is valuable as it can detect TR34 and L89H mutations from DNA samples faster than sequencing (Ahmad et al., 2014). The commercially available AsperGenius® (PathoNostics) can detect L98H, T289A, Y121F, and TR34 mutations as well as *A. fumigatus* gene directly from BALF specimen by multiplex real time PCR. In a multicenter clinical study, it showed good diagnostic performance on BAL and could detect *A. fumigatus* with resistance-associated mutations, including in culture-negative BALF samples, and detection of mutations was associated with azole treatment failure (Chong et al., 2016). However, the efficacy of this detection kit for CPA patients is unclear, as these mutations are relatively rare among patient-acquired azole-resistant strains obtained worldwide (Meis et al., 2016; Chowdhary et al., 2017); on the contrary, 27 (93.1%) of 29 of CPA patients from Europe had an L98H mutation from BALF samples and 16 (55.2%) had a TR34 mutation (Denning et al., 2011).

## Conclusion

**Figure 1:**
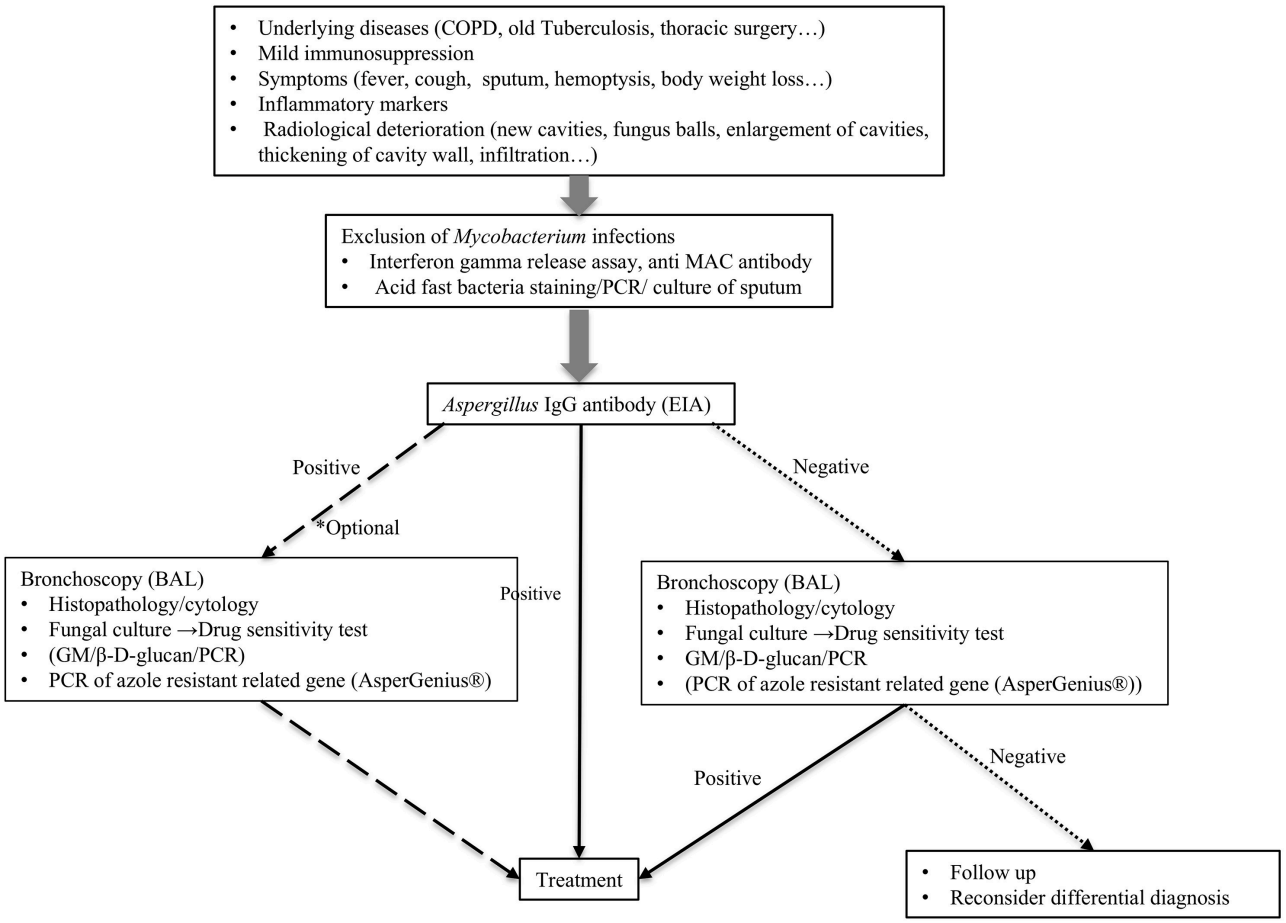
Proposed algorithm for the diagnosis of chronic pulmonary aspergillosis. BAL, bronchoalveolar lavage.

Needless to say, the gold standard of CPA diagnosis is the radiological findings of the lungs, its histopathology, and culture from the focus of infection. The definitive diagnosis by histopathology and culture is not always easy to perform; thereby, other diagnostic tools are also dispensable and biomarkers to reflect the disease status are needed. Diagnostic methods for CPA described in this review are summarized in Table 1. Currently, the *Aspergillus*-specific IgG antibody is the most promising tool for diagnosing CPA caused by *A. fumigatus*. We propose the algorithm for the diagnosis and treatment of CPA (Figure 1). When the patient is suspected of chronic aspergillus infection, it is important to rule out the mycobacterium infection first. Indication of bronchoscopy examination should be considered depending on the result of Aspergillus IgG antibody test. If it is negative, bronchoscopy examination is strongly recommended, as non-fumigatus Aspergillus infection can be the causative organism. If it is positive, bronchoscopy examination is however, optional, to determine which antifungal agents to be used, or collect more precise epidemiological information.

**Table 1:** Diagnostic methods for chronic pulmonary aspergillosis.

**Test**	**Specimen**	**Sensitivity (%)**	**Specificity (%)**	**Note**	**References**
Culture	Respiratory specimens	11.8–81.0	–		Kitasato et al., 2009; Kohno et al., 2010; Nam et al., 2010; Shin et al., 2014
β-D-glucan	Serum	15.4–26.7	95.8		Kitasato et al., 2009; Kohno et al., 2010; Urabe et al., 2017
	BALF	77.8	72.5	Cutoff ≧ 100 (Wako turbidimetric assay)	Urabe et al., 2017
Galactomannan	Serum	22.6–66.7	63.5	Cut off values differ in each study between 0.5 and 1.0	Kitasato et al., 2009; Kohno et al., 2010; Izumikawa et al., 2012; Shin et al., 2014
	BALF	77.2–77.8	77–90	Cut off values differ in each study between 0.4 and 0.5	Izumikawa et al., 2012; Urabe et al., 2017
*Aspergillus* precipitating antibody	Serum	56–89.3	100		Kitasato et al., 2009; Kohno et al., 2010; Baxter et al., 2013; Page et al., 2016
*Aspergillus* IgG antibody	Serum	93.2	98.2	Bio-Rad	Page et al., 2018
		83.8–98	84–98	ImmunoCAP	Fujiuchi et al., 2016; Page et al., 2016, 2018
		92.9–96	98–99.3	Immulite	Page et al., 2016, 2018
		84.2–90	91–98	Serion	Page et al., 2016, 2018
		77	97	Dynamiker	Page et al., 2016
		75	99	Genesis	Page et al., 2016
PCR	BALF	66.7–86.7	84.2–94.2	Non-standardized method	Urabe et al., 2017

*BALF, Bronchial alveolar lavage fluid*.

Since the emergence of azole-resistant *A. fumigatus* strains is a serious concern, convenient detection methods are required to detect these directly from clinical samples; however, further investigation is required. In addition, we need to investigate how these azole mutants are produced inside the lungs and how they affect CPA patients to discover other methods to decrease their prevalence.

## Author contributions

All authors listed have made a substantial, direct and intellectual contribution to the work, and approved it for publication.

### Conflict of interest statement

The authors declare that the research was conducted in the absence of any commercial or financial relationships that could be construed as a potential conflict of interest.

## Abbreviations

CCPA: chronic cavitary pulmonary aspergillosis
CFPA: chronic fibrosing pulmonary aspergilllosis
CT: Computed tomography
ABPA: Allergic bronchopulmonary aspergillosis
CPA: chronic pulmonary aspergillosis
BAL: bronchial alveolar lavage
BDG: β-D-glucan
GAG: galactosaminogalactan
GM: galactomannan
IPA: invasive pulmonary aspergillosis
LFD: lateral flow device
PCR: polymerase chain reaction
RT-PCR: Reverse transcription-polymerase chain reaction
SAIA: subacute invasive pulmonary aspergillosis
TR: tandem repeats.
